# On the Measurement of the Electron-Neutrino Correlation in Neutron Beta Decay

**DOI:** 10.6028/jres.110.061

**Published:** 2005-08-01

**Authors:** J. David Bowman

**Affiliations:** Los Alamos National Laboratory, Los Alamos, NM 87544, USA

**Keywords:** electron-neutrino correlation, neutron beta decay

## Abstract

I present a new approach to the measurement of *a*, the electron-neutrino correlation, in neutron beta decay. A precise measurement of *a* can lead to a precise determination of ratio of the axial vector and vector coupling constants, *G_a_/G_v_*. Coincidences between electrons and protons are detected in a field-expansion spectrometer. The field-expansion spectrometer is designed to make 1/*TOF* ≈ | *p*_p_|. *TOF* and *p*_p_ are the proton time of flight and momentum. Two segmented Si detectors view both electrons and protons in 4π geometry. The time of flight between the electron and proton are accurately measured in a long, ≈ 1 m, drift distance. The electron energy is accurately measured in the Si detectors. The proton momentum and electron energy determine the electron-neutrino opening angle. I have shown that by sorting the data on proton time of flight and electron energy, *a* can be determined with a statistical relative standard uncertainty of $\approx 2.4/\sqrt{n}$, where *n* is the number of decays observed. The approach has a number of advantages. The acceptance of the spectrometer is 4π for both particles. Thin-dead-layer segmented Si detectors as well as all other components in the apparatus, are commercially available. There are no material apertures to determine the acceptance of the apparatus. The charged particles interact only with electric and magnetic fields before striking the detectors. Coincident detection of electrons and protons reduces backgrounds, and allows the *in situ* determination of backgrounds. In the analysis, it is not necessary to sort on the relative electron and proton direction and hence electron back scattering does not cause systematic uncertainties. A time of flight spectrum is obtained for each electron energy. Different parts of the spectra have different sensitivities to *a*. The medium time of flight parts of the spectra that are insensitive to *a* can be used to verify the accuracy of the electric and magnetic field determinations.

## 1. Introduction and Discussion

**Fig. 1:**
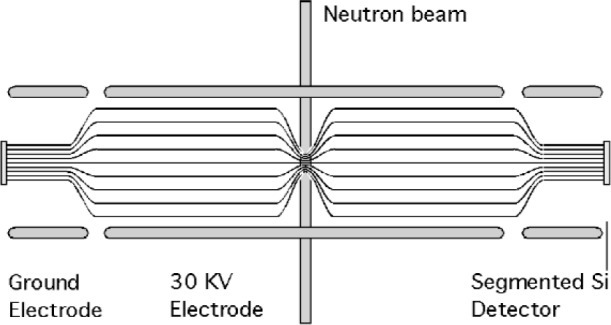
A schematic view of the field expansion spectrometer.

A reference design for the field expansion spectrometer is shown in Fig. 1. Electrons and protons spiral around magnetic field lines and are guided to two 10 cm by 10 cm segmented Si detectors. In the center of the spectrometer the field strength is 4 T, in the drift region 0.1 T, and near the Si detectors 1 T. The field expansion decreases the angle between the momentum and the magnetic field lines. The velocity in the drift region is close to |*p*_p_|/*m*, where *p*_p_ is the proton momentum and *m* is the proton mass. The particles strike the detectors at approximately normal angles and the probability of backscattering is reduced. An electric field is applied to the particles before they strike the Si detectors so that the protons have enough energy to be detected. The electric field reduces the energy of the electrons. The electric field must be applied after the magnetic field expansion so that the acceptance for electrons does not depend on electron energy. For the reference design, all electrons that have energies above 70 keV reach the detectors and deposit at least 30 keV. After the drift region the protons are accelerated from ≈ 400 eV to 30 keV so that the time spent between the potential change and the detector is small compared to the time spent in the drift region. Electrons may be scattered from the Si detectors, but scattered electrons are guided back to one of the detectors and eventually all the electrons’ energy is deposited in the detectors. The segmented Si detectors form an image of the beam. The ends of the decay region are defined by the image of the beam on the detectors. The transverse migration of back scattered electrons is small because the radius of gyration is small, a few mm, and because the momentum of the electron decreases with each reflection. I estimate that rates between 100 Hz and 1000 Hz can be obtained at the National Institute of Standards and Technology Center for Neutron Reseearch (NCNR) or the High Flux Isotope Reactor (HFIR) at Oak Ridge National Laboratory. A sample of 10^9^ events could be obtained in a 10^6^ s to 10^7^ s leading to a determination of *a* with a relative standard uncertainty of ≈ 0.1 %. The statistical relative standard uncertainty in *G_a_/G_v_* would be 0.0003 as compared to 0.003 in the Particle Data Listings [1].

The function of the spectrometer is to measure the magnitude of the proton momentum, *p*_p_. The electron neutrino correlation, *a*, expresses the dependence of decay rate on the angle between the neutrino and electron, cos(*ϑ*_ev_) = cos(*ϑ*_e_)cos(*ϑ*_v_) + sin(*ϑ*_e_)sin(*ϑ*_v_)cos(*φ*_e_ − *φ*_v_). *ϑ*e and *φ*_e_ are the electron polar and azimuthal angles, *ϑ*v and *φ*_v_ are the neutrino polar and azimuthal angles, and *ϑ*ev is the angle between the electron and neutrino. It is not necessary to measure all the above angles because *ϑ*_ev_ can be determined from the electron energy and the proton momentum squared. The electron energy is precisely measured in the Si detectors. The electron and neutrino momenta, *p*_e_ and *p*_v_, can be determined from the electron energy. cos(*ϑ*_ev_) can then be determined from the proton momentum and the electron energy using $p_{e}^{2}=p_{e}^{2}+2\;p_{e}p_{v}\cos\left(\vartheta ev\right)+p_{v}^{2}$. For a perfect determination of the proton momentum, *p*_p_ and cos(*ϑ*_ev_), the error in *a*, $\sigma_{a}=\sqrt{3/n\beta_{\text{ave}}^{2}}=2.3/\sqrt{n}$. $\beta_{\text{ave}}^{2}$ is the mean squared value of the electron velocity divided by the speed of light weighted by the phase space of the electron energy. The above reference design achieves $\sigma_{a}=2.4/\sqrt{n}$.

**Fig. 2:**
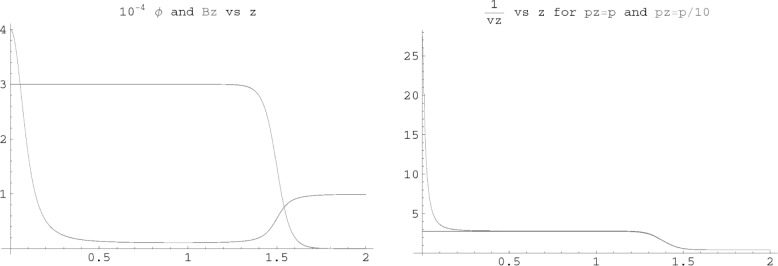
Plots of electric potential *ϕ*(*z*) and magnetic field *B_z_*(*z*), and the inverse proton velocity 1/*v_z_*(*z*) as functions of *z*. The horizontal axis for each plot is the distance from the center of the spectrometer in meters. The vertical axis on the left plot is the magnetic field strength in tesla or the electric potential in volts multiplied by 10^−4^. The vertical axis for the right plot is the inverse of the *z* component of the proton velocity in µs/m.

Figure 2 shows a plot of 1/*v_z_*(*z*) for two events with an electron energy of 391 keV. For one case the proton momentum is in the *z* direction and for the other with its momentum at at 84 degrees. The times of flight are 4.76 µs and 4.07 µs. The imperfect correspondence between *p*_p_ and *TOF* implies that the error in *a* will be larger than its theoretical minimum value. However, except for events where the proton momentum in the *z* direction is small, the correspondence is good. The best information on *G_a_/G_v_* has come from measurements of the electron-neutron spin correlation, *A*. In order to measure *A* it is necessary not only to determine the neutron polarization and but also which of the two detectors the electron struck first. This determination may be imperfect due to electron back scattering. The electron-neutrino opening angle depends on the square of the proton momentum and it is therefore not necessary to determine the relative direction of the electron and proton in order to measure the electron-neutrino correlation. The *TOF* and electron energy are sufficient. The practical implication of combining the two directions is important. It is possible to obtain commercially segmented Si detectors with thin ion-implanted entrance windows. The sheet resistance of the ion-implanted junction is large and the large rise time (≈ 50 ns) makes fast timing difficult. The ability to use slow Si detectors makes the experiment feasible without new technology.

**Fig. 3:**
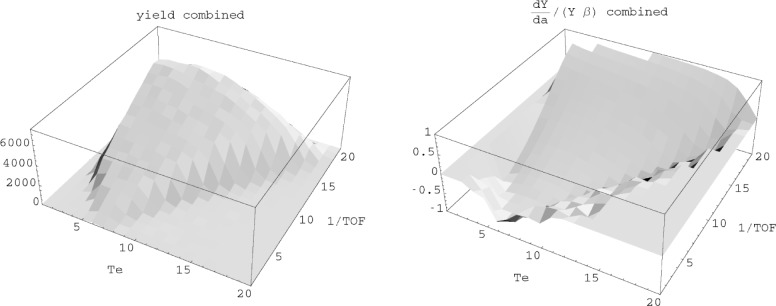
Yield and sensitivity for events binned on *T*_e_ and 1/*TOF*.

Figure 3 shows the results of a Monte-Carlo calculation of the error in *a*. Events were generated according to the electron kinetic energy and the angles of the electron and neutrino. The time of flight was calculated for the proton and using the adiabatic approximation. The yield and the logarithmic derivative of the yield with respect to *a* were calculated. The neutron decay rate is and ∼[1 + *a β* cos(*ϑ*_ev_)] and (1/*β*)(d ln(*Y*)/d*a*) approaches ±1 for small and large values of *TOF*, where cos(*ϑ*_ev_) = ±1, as expected.

The method discussed hear should be compared with the methods proposed at this meeting by Gordon Jones et al. [2] and S. Baessler et al [3]. The approach discussed here offers the advantages of 4π acceptance so that the number of events large leading to a smaller statistical error for a given running time. The present method offers advantages in the reduction of systematic errors as discussed above.

## 2. Conclusion

I have shown that a field-expansion spectrometer with a long drift distance to measure proton time of flight is an attractive approach to measure the electron-neutrino correlation in neutron beta decay. I emphasize the discussion of statistical and systematic errors, but a precise measurement of *a* will lead to the determination of *G_a_/G_v_*. The statistical effectiveness $\sigma =2.4/\sqrt{n}$ is favorable and the count rate is large. I estimate a statistical relative standard uncertainty of a few 10^−3^ could be obtained in a 10^6^ s to 10^7^ s run at HFIR or NIST. The approach avoids many of the potential sources of systematic uncertainty in other methods of determining *G_a_/G_v_*. It is not necessary to make a precise measurement of the neutron polarization. Backgrounds are greatly reduced by detecting electrons and protons in coincidence. The 4π acceptance of the spectrometer is well understood. The charged particles interact only with electric and magnetic fields before detection. Electron back scattering is not an issue, because it is not necessary to determine the relative directions of the electron and proton.
